# A Seed-Borne Bacterium of Rice, *Pantoea dispersa* BB1, Protects Rice from the Seedling Rot Caused by the Bacterial Pathogen *Burkholderia glumae*

**DOI:** 10.3390/life12060791

**Published:** 2022-05-26

**Authors:** Yusuke Kouzai, Chiharu Akimoto-Tomiyama

**Affiliations:** Crop Stress Management Group, Division of Plant Molecular Regulation Research, Institute of Agrobiological Sciences, National Agriculture and Food Research Organization (NARO), 2-1-2 Kannondai, Tsukuba 305-8602, Ibaraki, Japan; yusuke.k@affrc.go.jp

**Keywords:** rice, *Burkholderia glumae*, *Pantoea dispersa*, biocontrol, endophyte

## Abstract

Seedling rot, caused by the bacterial pathogen *Burkholderia glumae*, is a major disease of rice. It originates from pathogen-contaminated seeds and is thus mainly controlled by pesticide treatments of seeds. We previously demonstrated that the seed-borne bacteria of rice may be a useful and sustainable alternative to pesticides to manage seedling rot, but they are limited in terms of variety. Here, we report that another seed-borne bacterium, *Pantoea dispersa* BB1, protects rice from *B. glumae*. We screened 72 bacterial isolates from rice seeds of three genetically different cultivars inoculated or non-inoculated with *B. glumae*. 16S rRNA gene sequencing revealed that pathogen inoculation affected the composition of culturable seed-borne bacterial communities and increased the presence of *Pantoea* and *Paenibacillus* species. Among three *Pantoea* and *Paenibacillus* isolates that exhibit tolerance to toxoflavin, a virulence factor of *B. glumae*, *P. dispersa* BB1 significantly mitigated the symptoms of rice seedling rot. The culture filtrate of BB1 inhibited the growth of *B. glumae* in vitro, suggesting that this isolate secretes antibacterial compounds. Seed treatment with BB1 suppressed pathogen propagation in plants, although seed treatment with the culture filtrate did not. Because BB1 did not show pathogenicity in rice, our findings demonstrate that BB1 is a promising biocontrol agent against seedling rot.

## 1. Introduction

*Burkholderia glumae* is a soil-borne Gram-negative bacterium that causes seedling rot, sheath rot, and grain rot at the various stages of rice growth [1,2]. This pathogen threatens the global rice production, especially in East Asia, Central and South America, and in the United States [1]. The primary inoculum of seedling rot is represented by pathogen-contaminated seeds, and symptoms become apparent after germination [3,4]. The infected seedlings often rot and die in nursery boxes, leading to losses of planting materials. If the diseased seedlings are transplanted to fields, they become the secondary source of sheath and grain rot [5]. Therefore, the disease is mainly controlled by using pathogen-free seeds obtained via pesticide treatments. Treating seeds with oxolinic acid, an antibacterial quinone derivative, is highly effective to control *B. glumae*, but the development of oxolinic acid-resistant isolates of this pathogen has been reported in many fields in Japan [6]. Although a quantitative trait locus (QTL) for seedling rot resistance has been identified using chromosome segment substitution lines derived from a cross between Nona Bokra (indica landrace) and Koshihikari (japonica cultivar), no commercially available cultivars that exhibit complete resistance to *B. glumae* are currently known [7]. Moreover, high temperature and high humidity conditions favor the growth of this pathogen, and global warming could increase the risk of this rice disease in the future [1]. Considerable efforts are needed to develop effective methods to control seedling rot during rice production.

Toxoflavin is a bright yellow-colored phytotoxin produced by *B. glumae*, and it functions as a key virulence factor for rice seedling and grain rot [8]. The production of toxoflavin is regulated by quorum sensing; thus, mutations in the genes encoding the quorum sensing system reduce toxoflavin production by the pathogen, and its virulence towards rice [9]. Toxoflavin is also known to exhibit antifungal and antibacterial activities. It inhibits the growth of multiple genera of bacteria, including the seed-borne bacteria of rice [10], and has shown antifungal activity against a broad range of fungi, indicating its potential as a new fungicide [11].

Microbial biocontrol is an alternative or supplemental method adopted to reduce the use of pesticides and achieve sustainable agriculture [12]. Many microbes that can be used for crop disease management have been identified from plant environments such as the soil, rhizosphere, and endosphere [13]. For the control of *B. glumae*, a strain of *Burkholderia gladioli* isolated from rice seeds and its avirulent mutant could suppress the development of seedling rot and grain rot [14]. In addition, *Bacillus* sp., *Pseudomonas fluorescens*, and *Saccharomyces* sp. strains identified from a rice field showed antibacterial activity against *B. glumae* [1]. We previously demonstrated that four isolates of seed-borne bacteria of rice can improve rice seedling rot resistance. Seed treatment with three isolates of *Pseudomonas putida* (RSB1, 10, 15) suppresses rice growth disruption caused by *B. glumae* [10]. In contrast, *Stenotrophomonas maltophilia* RSB2 has no protective effect, but it can increase the protective effects of RSB1, 10, 15 by co-treatment, suggesting that rice seeds are promising sources of microbial biocontrol agents against seedling rot. However, the number of potential biocontrol agents that can be used practically in rice fields is still limited. In this study, we report *Pantoea dispersa* BB1 as a promising novel biocontrol agent against rice seedling rot.

## 2. Materials and Methods

### 2.1. Plant and Bacterial Materials

Three cultivars (Nipponbare [+/+], IRBB1, and TN1) of rice seeds, harvested in the test fields of the National Agricultural Research Organization (NARO, Tsukuba, Japan) in 2013, were used in this study. The seeds were stored in dry boxes at 4 °C until the experiments were performed. The *B. glumae* strain MAFF301682 was obtained from Genebank at NARO and was cultured on LB medium at 30 °C.

### 2.2. Inoculation Tests

*B. glumae* MAFF301682 was cultured in LB liquid medium at 30 °C overnight and was then collected by centrifugation. The collected bacteria were resuspended in sterile distilled water (SDW), washed three times, and then the concentration of the bacterial suspension was adjusted to the indicated optical density of 600 nm (OD_600_) and used for inoculation. Rice seeds were surface-disinfected with a sodium hypochlorite solution (WAKO, Tokyo, Japan) and were incubated in SDW at 30 °C for 3 days in the dark. Then, they were vacuum-inoculated with the bacterial suspension for 5 min and were agitated at normal pressure for 30 min. The inoculated seeds were transferred to sterilized soil (Bonsol No. 2, Sumitomo Chemical, Tokyo, Japan) and were grown in a growth chamber at 28 °C with 100% humidity under a 16 h:8 h photoperiod. Disease severity was evaluated by measuring the shoot length of the inoculated plants, as previously described [10].

### 2.3. Isolation and Characterization of the Seed-Borne Bacteria of Rice

Rice seeds were inoculated with *B. glumae*, as described above, and were collected at 4 days post inoculation (dpi). The collected plants were washed with SDW three times and crushed using a Micro Smash bead beater (Tomy, Tokyo, Japan) with 1 mL of SDW and two stainless-steel beads (4.8 mm). The plant extract was serially diluted with SDW, transferred to LB agar plates, and was then incubated for 3 to 4 days at 30 °C until bacterial colonies were visually observed. Each morphological distinct colony was randomly selected and stored in 20% glycerol solution at −80 °C. To characterize the isolated bacteria, bacterial DNA was extracted using the DNeasy Blood &Tissue Kit (QIAGEN, Hilden, Germany), and PCR for the bacterial 16S rRNA gene was performed using universal primers. The PCR conditions were described previously [10], and the primer sequences are listed in Table S1. The PCR fragments were sequenced using the BigDye Terminator v3.1 Cycle Sequencing Kit (Thermo Fisher Scientific, Waltham, MA, USA) and a SeqStudio Genetic Analyzer (Thermo Fisher Scientific), and they were annotated via the BLAST web service of the DNA Data Bank of Japan. For phylogenetic analysis, the 16S rRNA gene sequences of *Pantoea* species were retrieved from SILVA (https://www.arb-silva.de/, accessed on 17 January 2022). The selection of *Pantoea* species was based on previous reports [15]. The sequences were aligned with MUSCLE software (http://www.drive5.com/muscle/, accessed on 17 January 2022), and a phylogenetic tree was constructed using the neighbor-joining method in MEGA software (https://www.megasoftware.net/, accessed on 17 January 2022) [16].

### 2.4. Toxoflavin Tolerance Tests

Each bacterial isolate was cultured in LB liquid medium at 30 °C overnight; bacterial cells were then collected by centrifugation, washed with SDW three times, and resuspended with fresh LB liquid medium. Subsequently, 100 µL of bacterial suspension (OD_600_ of 0.1) was transferred to a 96-well microplate and incubated with or without 0.1 mM toxoflavin (also referred to as PKF118-310, Merck, Kenilworth, NJ, USA) at 30 °C with shaking. The bacterial growth was measured using an Infinite 200PRO microplate reader (TECAN, Männedorf, Switzerland).

### 2.5. Antibacterial and Biocontrol Analyses of the Culture Filtrate

*P. dispersa* BB1 was cultured in LB liquid medium at 30 °C until the OD_600_ exceeded 2.0; then, the cells were removed by centrifugation (8000 rpm, 5 min) and the supernatant was passed through a 0.22-μm-pore syringe filter (Sartorius, Göttingen, Germany) in order to obtain the culture filtrate. For antibacterial analysis, the overnight culture of *B. glumae* was collected by centrifugation, washed with SDW three times, and resuspended with fresh LB liquid medium. Then, 100 µL of the *B. glumae* suspension (OD_600_ of 0.1) was transferred to a 96-well microplate and incubated with or without 100 µL of the culture filtrate diluted with SDW (to the final concentrations of 5, 25, or 50% *v/v*) at 30 °C. Bacterial growth was measured using an Infinite 200PRO microplate reader (TECAN). For biocontrol analysis, the *B. glumae* suspension (OD_600_ of 0.008) was mixed with an equal volume of the culture filtrate to prepare the inoculants. Surface-disinfected rice seeds were vacuum-inoculated with the inoculants, transferred to sterilized soil (Bonsol No. 2, Sumitomo Chemical), and were grown in a growth chamber at 28 °C with 100% humidity under a 16 h:8 h photoperiod. Disease severity was evaluated by measuring shoot length and pathogen propagation in the inoculated plants. In these, the *B. glumae* biomass in the inoculated plants was quantified using qPCR based on procedures described in previous reports [17,18,19]. qPCR was performed using the SYBR Premix Ex Taq II (Takara Bio, Shiga, Japan) with a TAKARA PCR Thermal Cycler Dice (Takara Bio) according to the manufacturer’s instructions. The rice ubiquitin gene was used for normalization. Primers are listed in Table S1.

## 3. Results

### 3.1. Isolation of Seed-Borne Bacteria

**Figure 1:**
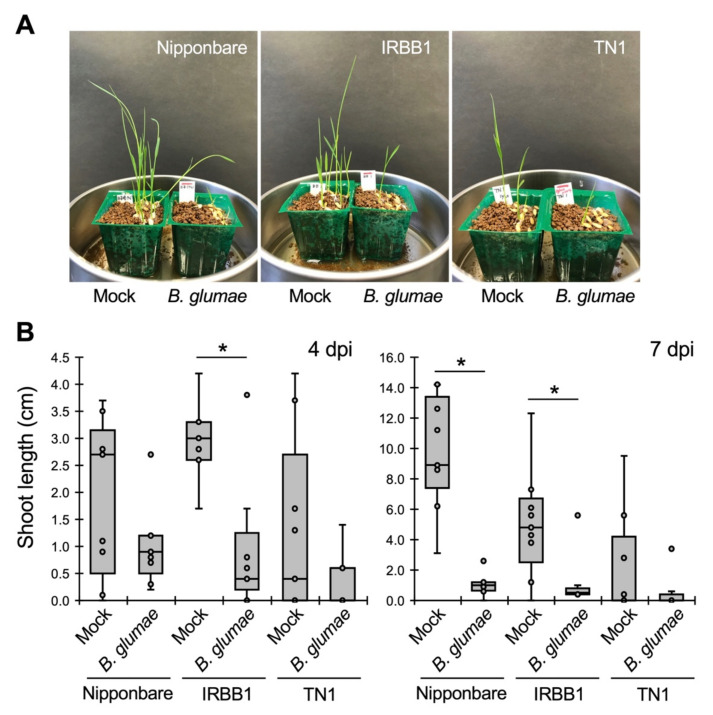
Pathogenicity of *B. glumae* for three genetically different rice cultivars. Rice seeds of Nipponbare, IRBB1, and TN1 were inoculated with *B. glumae* (OD_600_ of 0.004) and with sterilized distilled water (SDW, mock). (**A**) Disease symptoms at 7 days post inoculation (dpi). (**B**) Box plots of the shoot length of inoculated plants at 4 and 7 dpi. The center line indicates the median, the box limits are the upper and lower quartiles, the whiskers indicate the maximum and minimum values, and the representative data are indicated as dots. Asterisks (*) represent statistically significant differences comparing the values of mock and *B. glumae* inoculation (Student’s *t*-tests, *p* < 0.05, *n* = 9). The experiments were repeated twice with similar results.

We used three genetically different rice cultivars (Nipponbare, IRBB1, and TN1) harvested in the same field in Japan. Nipponbare is a genome-sequenced standard japonica cultivar, whereas IRBB1 is an indica cultivar carrying *Xa1*, which is a bacterial blight resistance gene. TN1, also called Taichung Native 1, is an indica cultivar widely used in disease susceptibility tests. We inoculated the seeds of these cultivars with *B. glumae* and found that their growth was strongly inhibited (Figure 1), suggesting that the cultivars are susceptible to this pathogen. Compared with Nipponbare and IRBB1, TN1 showed reduced germination and growth, regardless of pathogen inoculation (Figure 1). This may depend on the conditions of the seeds used in the experiment.

**Figure 2:**
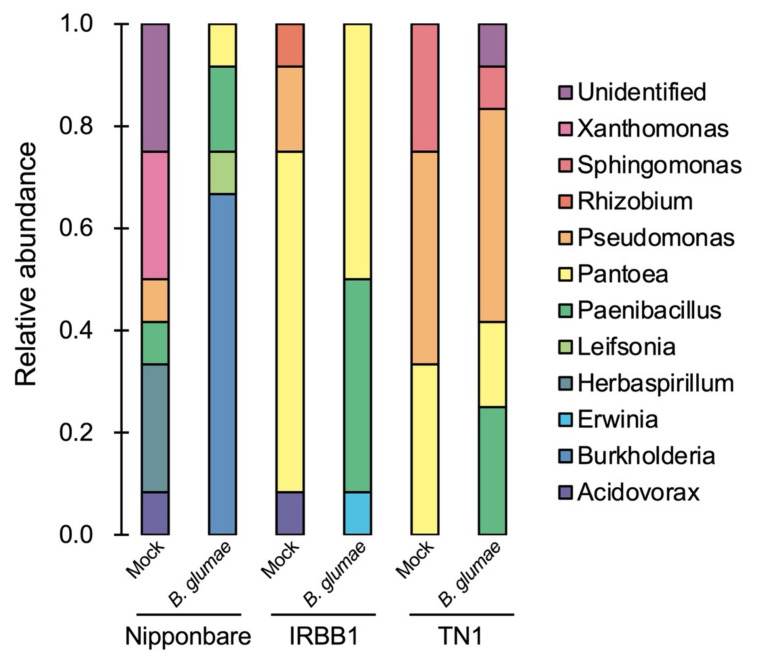
Composition of the culturable bacterial communities isolated from rice seeds inoculated or non-inoculated with *B. glumae*. Seventy-two bacterial isolates were isolated from rice seeds inoculated with SDW (mock) or *B. glumae* by serial dilution plating. Their bacterial genera were identified by 16S rRNA sequencing, and the relative abundance of each genus is represented as stacked bar graphs.

We isolated seed-borne bacteria from the rice plants inoculated with *B. glumae* or SDW (mock) at 4 dpi by serial dilution plating using LB medium. A total of 72 bacterial colonies were simply identified by 16S rRNA gene sequencing and were classified into 11 genera (Table S2). In all three cultivars, the isolated bacterial genera were different between mock- and pathogen-inoculated plants (Figure 2), indicating that the *B. glumae* infection affects seed-borne bacterial communities. While the *Acidoborax*, *Herbaspirillum*, *Rhizobium*, and *Xanthomonas* genera were identified only in mock-inoculated plants, *Burkholderia*, *Erwinia*, and *Leifsonia* were specific to *B. glumae*-inoculated plants. *Burkholderia*, which includes *B. glumae*, was detected in Nipponbare, but not in IRBB1 and TN1. *Paenibacillus*, *Pantoea*, *Pseudomonas*, and *Sphingomonas* were common in mock- and *B. glumae*-inoculated plants. Of these, *Paenibacillus* and *Pantoea* were present in the inoculated plants of all three cultivars and were particularly abundant in IRBB1 and TN1. These results suggest that the isolates of these two genera have the potential to colonize infected rice tissues.

### 3.2. Screening of Biocontrol Bacteria

**Figure 3:**
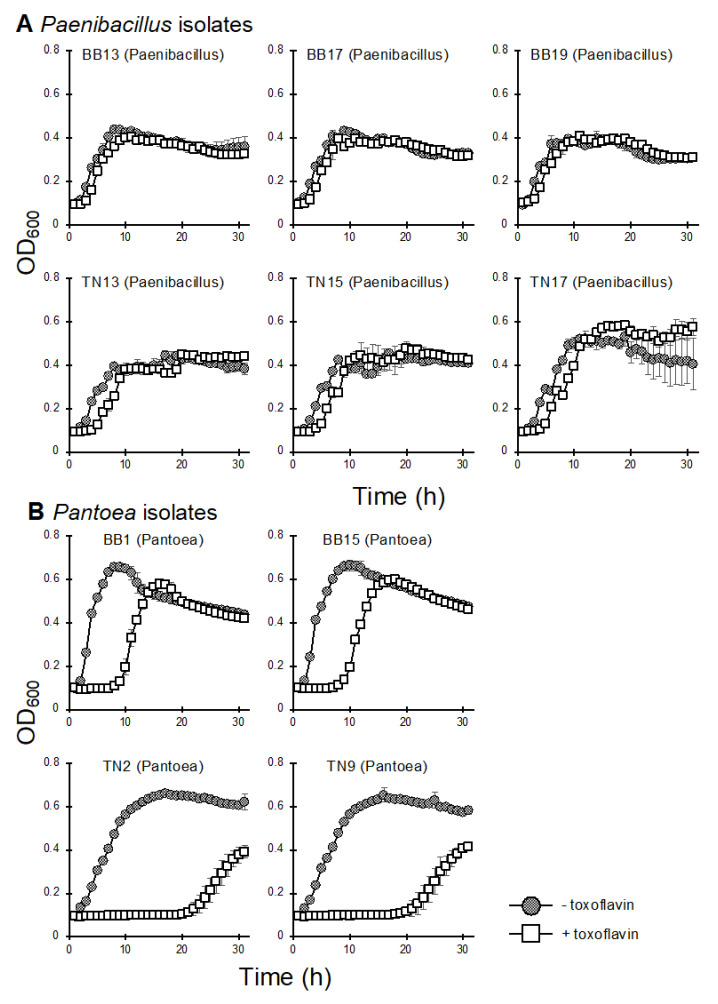
Bacterial growth in liquid LB medium containing toxoflavin. The (**A**) *Paenibacillus* and (**B**) *Pantoea* isolates derived from IRBB1 and TN1 rice seeds were cultivated in liquid LB medium containing 0.1 mM toxoflavin. The growth of each isolate was measured as OD_600_ every hour. Error bars represent standard deviation (*n* = 3). The experiments were repeated twice with similar results.

To explore seed-borne bacteria that have biocontrol activity, we conducted a toxoflavin tolerance test. As toxoflavin is known to have antibacterial activity [10], the bacterial biocontrol agents against *B. glumae* may need to exhibit tolerance to this toxin. We examined the 10 IRBB1- and TN1-derived isolates of *Pantoea* and *Paenibacillus* and found that the latter were highly tolerant to toxoflavin (Figure 3A). The *Pantoea* isolates showed a certain level of toxoflavin tolerance (Figure 3B) compared with the *Herbaspirillum* and *Acidovorax* isolates derived from mock-inoculated Nipponbare plants (Figure S1). However, interestingly, the *Pantoea* isolates derived from TN1 were more sensitive to toxoflavin than those derived from IRBB1. We then selected the *Pantoea* isolate BB1 and *Paenibacillus* isolates BB17 and BB19 for further analysis.

**Figure 4:**
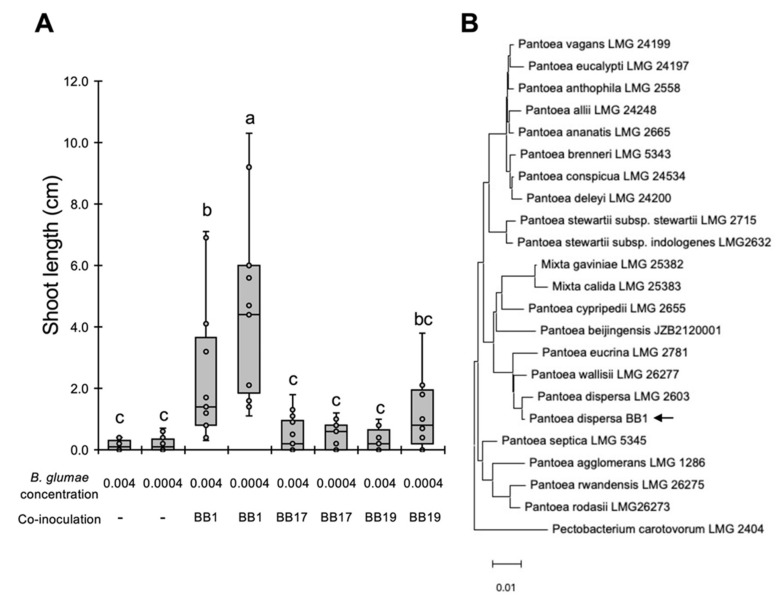
*P. dispersa* BB1 recovers rice growth disruption caused by *B. glumae*. (**A**) Rice seeds of Nipponbare were inoculated with *B. glumae* (OD_600_ of 0.004 or 0.0004) and the BB1, BB17, and BB19 isolates (OD_600_ of 0.004). Box plots indicate the shoot length of inoculated plants at 7 dpi. The center line indicates the median, the box limits are the upper and lower quartiles, the whiskers indicate the maximum and minimum values, and the representative values are indicated as dots. Different letters indicate statistically significant differences between groups (Tukey’s HSD test, *p* < 0.05, *n* = 17). The experiments were repeated twice with similar results. (**B**) Phylogenetic analysis of isolate BB1 based on 16S rRNA sequences of selected *Pantoea* species. Sequence alignment was performed in MUSCLE, and the tree was constructed via the neighbor-joining method in MEGA X. The arrow indicates *P. dispersa* BB1. The scale bar represents 0.01% divergence. *Pectobacterium carotovorum* LMG2404 was used as an outer group.

To examine whether BB1, BB17, and BB19 can protect rice from *B. glumae*, Nipponbare seeds were co-inoculated with *B. glumae* and each isolate. Whereas co-inoculation with the *Pantoea* isolate BB1 significantly suppressed rice growth inhibition, that with *Paenibacillus* isolates BB17 and BB19 did not at two levels of inoculum pressure (Figure 4A), indicating that only BB1 has biocontrol activity. We then tested the pathogenicity of BB1 to rice and found that this isolate does not impair rice growth at the seedling stage (Figure S2). To determine the species of BB1, almost the full length of the 16S rRNA gene was sequenced, and a blast analysis revealed that the sequence obtained was closely related to those of *P. dispersa* LMG 2603 (Identity: 99.85%, E-value: 0.0) and *P. dispersa* DSM 30073 (Identity: 99.31%, E-value: 0.0). Moreover, BB1 was classified into the same clade of *P. dispersa* LMG 2603, based on a phylogenetic analysis using 20 species belonging to the genus *Pantoea* (Figure 4B). These results demonstrate that BB1, identified as *P. dispersa*, can suppress the symptoms of seedling rot caused by *B. glumae*.

### 3.3. Biocontrol Mechanisms of P. dispersa BB1

**Figure 5:**
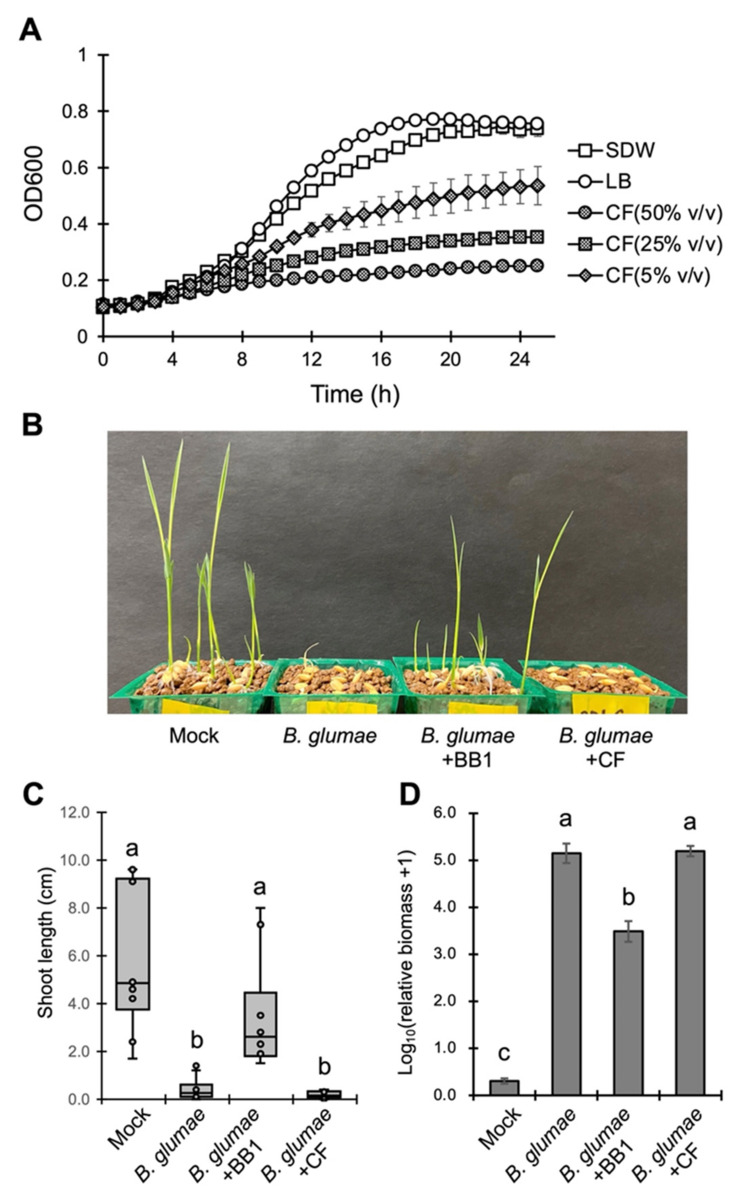
The *P. dispersa* BB1 culture filtrate shows antibacterial activity against *B. glumae*. (**A**) Growth of *B. glumae* cultured in liquid LB medium containing the culture filtrate of *P. dispersa* BB1 (CF). The growth of *B. glumae* was measured as OD_600_ every hour. Error bars represent standard deviation (*n* = 4). (**B**) Disease symptoms of rice plants inoculated with SDW (mock), *B. glumae*, BB1, and CF at 7 dpi. (**C**) Box plot of the shoot length of inoculated plants at 7 dpi. The center line indicates the median, the box limits are the upper and lower quartiles, the whiskers indicate the maximum and minimum values, and the representative values are indicated as dots. Different letters indicate statistically significant differences between groups (Tukey’s HSD test, *p* < 0.05, *n* = 10). (**D**) Relative biomass of *B. glumae* in the inoculated plants at 7 dpi. Data are presented as means ± SEM of values relative to mock-inoculated plants. Different letters indicate statistically significant differences between groups (Tukey’s HSD test, *p* < 0.05, *n* = 4). These experiments were repeated in triplicates with similar results.

To understand how *P. dispersa* BB1 protects rice from *B. glumae*, we evaluated the antibacterial and biocontrol activity of the culture filtrate of BB1. Biocontrol bacteria often produce and secrete compounds that show antibacterial activity or reduce the virulence of pathogens [20]. We prepared the culture filtrate of BB1 and found that it inhibited the growth of *B. glumae* in vitro in a concentration-dependent manner (Figure 5A). However, it could not suppress disease symptoms and pathogen propagation in the inoculated plants (Figure 5B–D). These results indicate that the BB1 culture filtrate exhibits antibacterial, but not biocontrol, activity against *B. glumae*. In contrast, BB1 itself significantly reduced pathogen propagation in the inoculated plants (Figure 5D), suggesting that it inhibits the growth of *B. glumae* in rice plants and, consequently, suppresses the symptoms of seedling rot.

## 4. Discussion

The present study demonstrated that the *P. dispersa* BB1 isolated from rice seeds reduces the disease symptoms caused by *B. glumae* in rice (Figure 4). The bacterial genus *Pantoea* comprises yellow-colored Gram-negative bacteria that are frequently isolated from plant, animal, soil, and water environments [15]. In the context of plant–microbe interactions, *Pantoea* is primarily recognized as a pathogenic genus. For example, *P. ananatis* can infect a broad range of monocot and dicot plants, including economically important crops [21], and it caused severe yield losses in onion production as the causal agent of center rot in South Korea [22]. *P. dispersa* is also reported as a pathogen threatening rice and onion production in East Asia [23,24]. However, some *Pantoea* strains have traits that are beneficial for plants. *P. ananatis* BLBT1-08 is an avirulent strain identified from healthy grape berries, and it can protect grapevines from the necrotrophic fungal pathogen *Botrytis cinerea* [25]. *P. dispersa* VWB2, which is isolated from rice seeds, promotes host growth by producing the phytohormone auxin [26]. Moreover, *P. dispersa* strains isolated from healthy sweet potato show biocontrol activity against the fungal pathogen *Ceratocystis fimbriata* in sweet potato plants [27]. These reports suggest that the *Pantoea* species living in plant environments could, opportunistically, be both pathogenic and biocontrol agents. Our results demonstrate that *P. dispersa* BB1 did not show pathogenicity against rice at the seedling stage (Figure S2), highlighting its potential as a biocontrol agent to limit seedling rot.

Several mechanisms through which microbial biocontrol agents protect plants from pathogens have been reported, such as competition for nutrients, production of antimicrobial compounds, and induction or priming of host disease resistance [13]. We demonstrated that BB1 suppressed the growth of *B. glumae* and the symptoms of seedling rot in planta, and that its culture filtrate showed antibacterial activity against *B. glumae* in vitro (Figure 5). These results imply that BB1 restricts the growth of *B. glumae* in rice plants through the production of antibacterial compounds. It has been reported that a broad range of biocontrol bacteria produce such compounds, which include small organic molecules, lipopeptides, and lytic enzymes [12]. Some *Pantoea* strains have also been shown to secrete small antimicrobial compounds, such as the peptide-derived Pantocin A [28], and a natural product known as PNP-1 [29]. However, the culture filtrate of BB1 itself could not suppress the growth of *B. glumae* and the development of seedling rot in rice plants (Figure 5C,D). Although the production of antimicrobial compounds in vitro has often been explained as a mode of action of biocontrol bacteria, it is not well understood whether these compounds are produced at the site where pathogens and biocontrol bacteria interact [13]. In plant and soil environments, antimicrobial compounds produced by biocontrol agents may have a short half-time or may not diffuse efficiently [30,31]. Therefore, future studies should compare the amount and diffusion of the compounds produced by BB1 between in vitro and in planta conditions; however, at this time, we cannot exclude the possibility that BB1 suppresses the growth of *B. glumae* in planta by mechanisms other than antibacterial compounds. The identification of the genes encoding the production of these substances can contribute to clarifying the biocontrol mechanisms of BB1.

Pathogenic infections, as well as damage to tissues, are known to modify plant microbiota [32]. In mock-inoculated plants, the isolated bacterial genera differed among the three rice cultivars in our experimental conditions, even though these were harvested in the same field at the same time (Figure 2). Similarly, the endophytic microbial communities of seeds were shaped by plant genotype in indica rice and domesticated wheat cultivars [33,34]. These findings indicate that plant genetic factors may be one of the determinants of the structures of rice seed microbiota, in addition to soil and environmental conditions. However, in pathogen-inoculated plants, the *Paenibacillus* and *Pantoea* isolates were frequently detected in all cultivars (Figure 2), and one of the *Pantoea* isolates, BB1, could protect rice plants from *B. glumae* (Figure 4). These findings suggest that pathogen infection or inoculation may induce the colonization of endophytic bacteria that are beneficial for disease resistance in host plants. Similarly, inoculation with the pathogenic fungus *Fusarium graminearum* was shown to modify the rhizosphere microbiota in barley plants, and bacterial taxa carrying antifungal traits are selectively recruited upon pathogen infection [35]. In chili pepper, infection with the soil-borne fungal pathogen *Fusarium oxysporum* affects the microbial interkingdom network and recruits plant-beneficial bacteria in the aboveground parts of diseased plants [36]. The comparison of plant microbiota between healthy and diseased plants and the isolation of plant-associated microbes from diseased tissues may represent effective strategies to identify novel biocontrol agents.

IRBB1 and TN1 were found to be susceptible to *B. glumae*, even though the *Pantoea* isolates were frequently detected in mock-inoculated plants (Figure 1 and Figure 2). There are two possible reasons for this complicated point. First, the composition of culturable seed-borne bacterial communities may not reflect the actual composition. We analyzed only bacterial isolate that could be isolated by serial dilution plating using LB medium. Therefore, the abundance of the *Pantoea* isolates may be much smaller in the actual communities. 16S rRNA amplicon sequencing needs to infer the actual composition of seed-borne bacterial communities in IRBB1 and TN1. Second, all the *Pantoea* isolates identified in this study may not necessarily have biocontrol activity. The previous report showed that, of the 12 *Pantoea* isolates isolated from sweet potato, only four showed antifungal activity against *C. fimbriata* [27]. In field trials, Nipponbare was susceptible to *B. glumae* [7], but it is unclear whether IRBB1 and TN1 are also susceptible. Combination with 16S rRNA amplicon sequencing and field trials is required to clarify whether the abundance of *Pantoea* species in rice seeds correlates with host resistance to *B. glumae*.

The present study identified a novel biocontrol agent against rice-pathogenic *B. glumae* in rice. In combination with our previous report [10], at least five isolates of seed-borne bacteria of rice were found to be involved in the suppression of seedling rot. The monitoring of endophytic bacterial communities in rice could be useful to predict the development of seedling rot and to elucidate the mechanism of opportunistic infection by *B. glumae*. Through the action of toxoflavin, this pathogen causes disease symptoms, not only in rice, but also in other field crops, such as tomato, sesame, and eggplant [8]. Future studies are needed to determine whether *P. dispersa* BB1 can control infections caused by *B. glumae* in other economically important crops.

## Data Availability

Not applicable.

## Supplementary Materials

The following supporting information can be downloaded at: https://www.mdpi.com/article/10.3390/life12060791/s1, Figure S1: Growth of the bacterial isolates from mock-inoculated plants in liquid LB medium containing toxoflavin; Figure S2: Growth of rice plants after inoculation with *P. dispersa* BB1; Table S1: Primers used in this study; Table S2: Bacterial isolates from genetically different three cultivars of rice seeds.
