# Supplementary material for: A Seed-Borne Bacterium of Rice, Pantoea dispersa BB1, Protects Rice from the Seedling Rot Caused by the Bacterial Pathogen Burkholderia glumae
Source: Life (Basel). 2022 May 26;12(6):791. doi: 10.3390/life12060791 (PMC9225591; doi:10.3390/life12060791)
Supplement: Supplementary file 1 [file life-12-00791-s001.zip › Figures S1 and S2_Kouzai_Akimoto.pptx]

## Slide 1
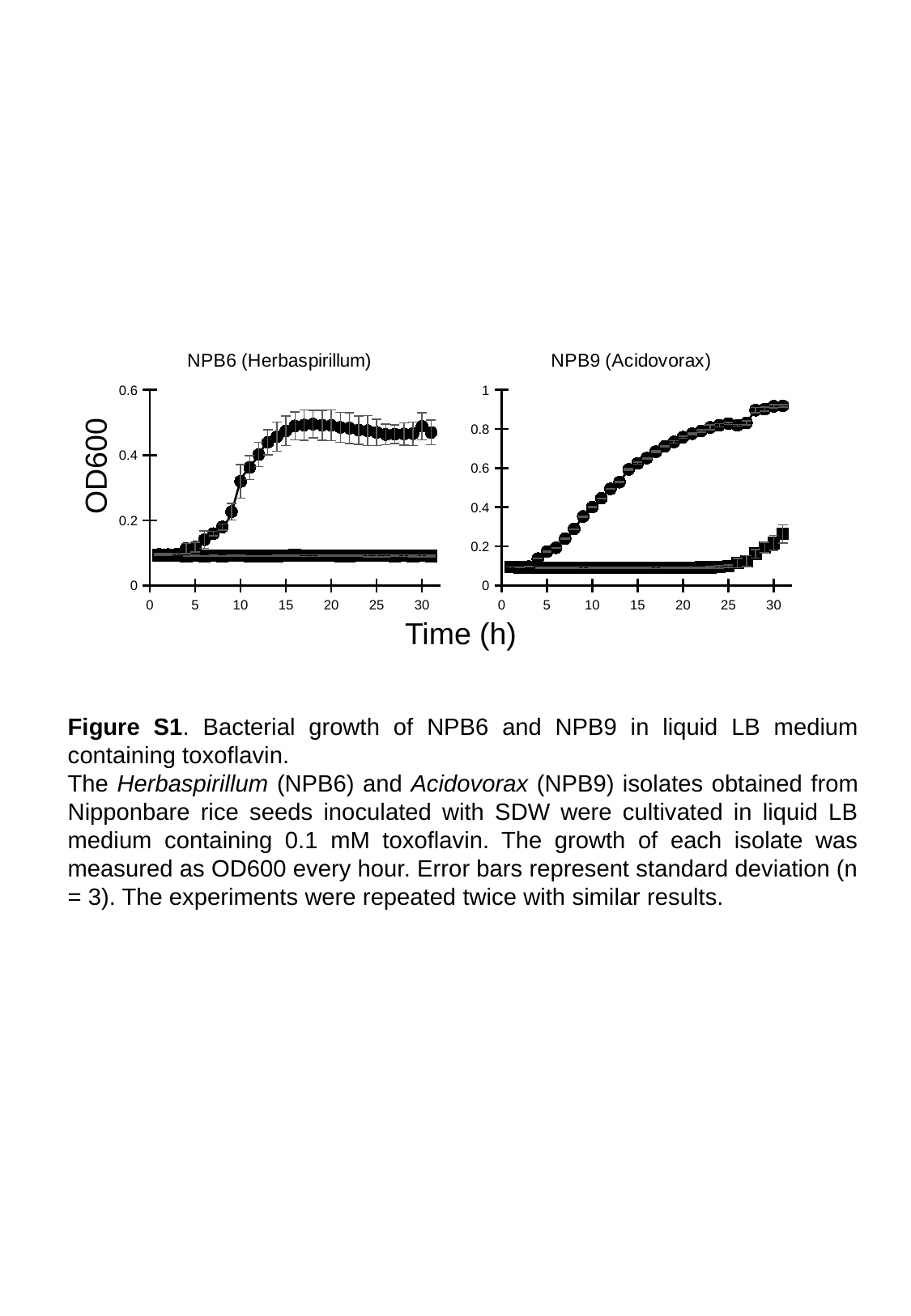

### Chart: NPB6 (Herbaspirillum)
| Category | -Tox | +Tox |
|---|---|---|
### Chart: NPB9 (Acidovorax)
| Category | -Tox | +Tox |
|---|---|---|OD600
Time (h)
Figure S1. Bacterial growth of NPB6 and NPB9 in liquid LB medium containing toxoflavin.
The Herbaspirillum (NPB6) and Acidovorax (NPB9) isolates obtained from Nipponbare rice seeds inoculated with SDW were cultivated in liquid LB medium containing 0.1 mM toxoflavin. The growth of each isolate was measured as OD600 every hour. Error bars represent standard deviation (n = 3). The experiments were repeated twice with similar results.

## Slide 2
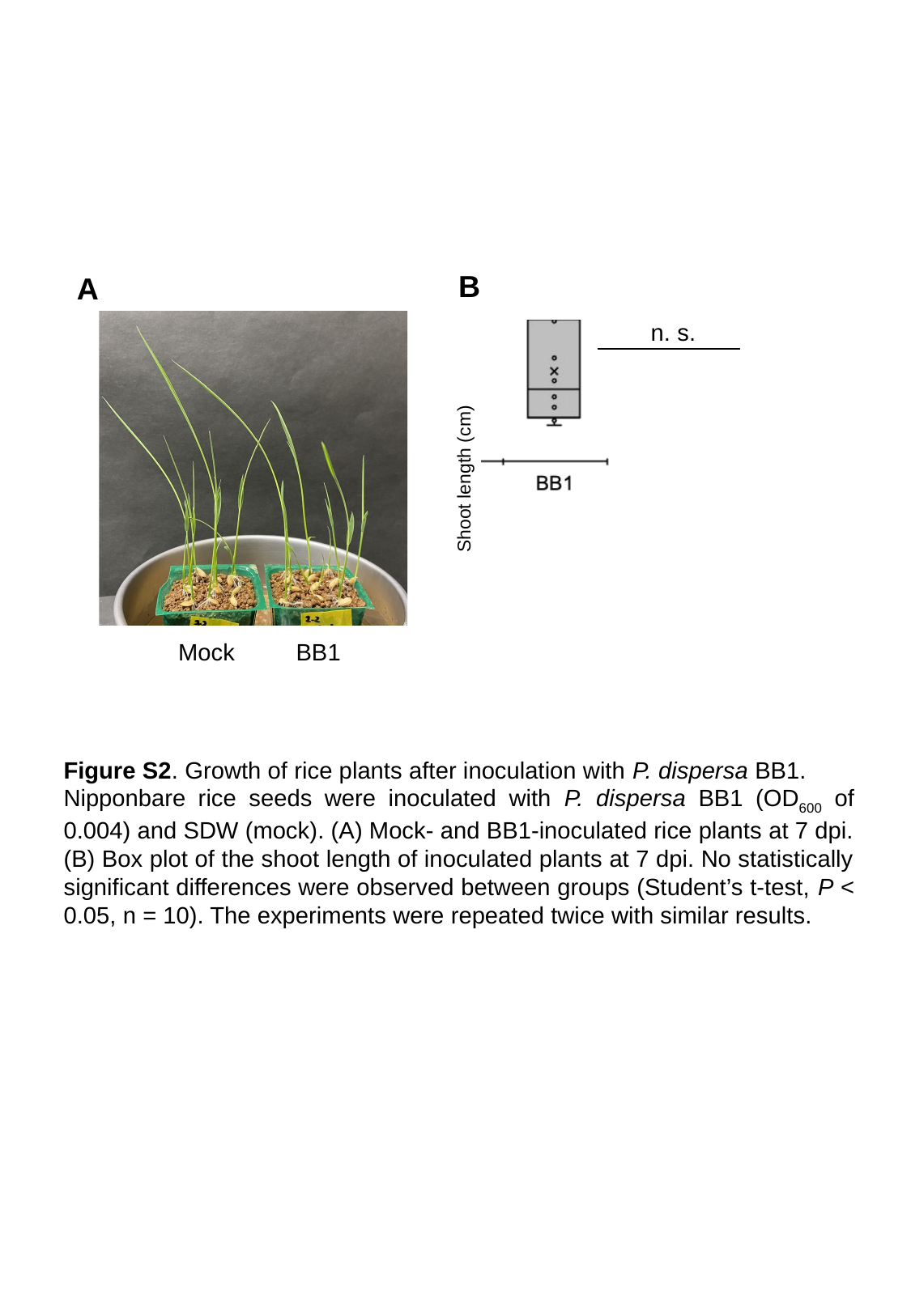

B
A
n. s.
Shoot length (cm)
Mock
BB1
Figure S2. Growth of rice plants after inoculation with P. dispersa BB1.
Nipponbare rice seeds were inoculated with P. dispersa BB1 (OD600 of 0.004) and SDW (mock). (A) Mock- and BB1-inoculated rice plants at 7 dpi. (B) Box plot of the shoot length of inoculated plants at 7 dpi. No statistically significant differences were observed between groups (Student’s t-test, P < 0.05, n = 10). The experiments were repeated twice with similar results.
